# Evaluating Clinical Genome Sequence Analysis by Watson for Genomics

**DOI:** 10.3389/fmed.2018.00305

**Published:** 2018-11-09

**Authors:** Kota Itahashi, Shunsuke Kondo, Takashi Kubo, Yutaka Fujiwara, Mamoru Kato, Hitoshi Ichikawa, Takahiko Koyama, Reitaro Tokumasu, Jia Xu, Claudia S. Huettner, Vanessa V. Michelini, Laxmi Parida, Takashi Kohno, Noboru Yamamoto

**Affiliations:** ^1^Department of Experimental Therapeutics, National Cancer Center Hospital, Tokyo, Japan; ^2^Department of Hepatobiliary and Pancreatic Oncology, National Cancer Center Hospital, Tokyo, Japan; ^3^Division of Translational Genomics, National Cancer Center-Exploratory Oncology Research and Clinical Trial Center, Tokyo, Japan; ^4^Department of Bioinformatics, National Cancer Center Research Institute, Tokyo, Japan; ^5^Department of Clinical Genomics, National Cancer Center Research Institute, Tokyo, Japan; ^6^IBM T. J. Watson Research Center, Yorktown Heights, NY, United States; ^7^Tokyo Software & Systems Development Laboratory, IBM Japan, Tokyo, Japan; ^8^IBM Watson Health, Cambridge, MA, United States; ^9^Division of Genome Biology, National Cancer Center Research Institute, Tokyo, Japan

**Keywords:** clinical genome sequencing, genome sequencing interpretation, artificial intelligence, watson for genomics, precision medicine

## Abstract

**Background:** Oncologists increasingly rely on clinical genome sequencing to pursue effective, molecularly targeted therapies. This study assesses the validity and utility of the artificial intelligence Watson for Genomics (WfG) for analyzing clinical sequencing results.

**Methods:** This study identified patients with solid tumors who participated in in-house genome sequencing projects at a single cancer specialty hospital between April 2013 and October 2016. Targeted genome sequencing results of these patients' tumors, previously analyzed by multidisciplinary specialists at the hospital, were reanalyzed by WfG. This study measures the concordance between the two evaluations.

**Results:** In 198 patients, in-house genome sequencing detected 785 gene mutations, 40 amplifications, and 22 fusions after eliminating single nucleotide polymorphisms. Breast cancer (*n* = 40) was the most frequent diagnosis in this analysis, followed by gastric cancer (*n* = 31), and lung cancer (*n* = 30). Frequently detected single nucleotide variants were found in *TP53* (*n* = 107), *BRCA2* (*n* = 24), and *NOTCH2* (*n* = 23). *MYC* (*n* = 10) was the most frequently detected gene amplification, followed by *ERBB2* (*n* = 9) and *CCND1* (*n* = 6). Concordant pathogenic classifications (i.e., pathogenic, benign, or variant of unknown significance) between in-house specialists and WfG included 705 mutations (89.8%; 95% CI, 87.5%−91.8%), 39 amplifications (97.5%; 95% CI, 86.8–99.9%), and 17 fusions (77.3%; 95% CI, 54.6–92.2%). After about 12 months, reanalysis using a more recent version of WfG demonstrated a better concordance rate of 94.5% (95% CI, 92.7–96.0%) for gene mutations. Across the 249 gene alterations determined to be pathogenic by both methods, including mutations, amplifications, and fusions, WfG covered 84.6% (88 of 104) of all targeted therapies that experts proposed and offered an additional 225 therapeutic options.

**Conclusions:** WfG was able to scour large volumes of data from scientific studies and databases to analyze in-house clinical genome sequencing results and demonstrated the potential for application to clinical practice; however, we must train WfG in clinical trial settings.

## Introduction

Recent progress in precision medicine applied to cancer therapy, i.e., targeted treatment based on the specific molecular features of a patient's tumor has changed cancer treatment strategies (1, 2). With increasing frequency, oncologists are sequencing tumor genomes in clinical practice to identify molecularly targeted treatment options (3). Next-generation sequencing is a powerful tool for building a tumor-specific profile of genomic alterations, such as single nucleotide variants, copy number variants, and gene translocations. The challenge lies in identifying which mutations are pathogenic and actionable. This is generally done by teams of human experts who reference databases of genomic alterations, published literature, regulatory approval data for pharmaceutical agents, and clinical trial protocols to identify patients who may benefit from treatment by molecularly targeted therapies (4, 5). This translation of genome sequencing results into potential treatment options is time-consuming, laborious, and requires a high degree of specialization, but the growing list of targeted therapies is making it more and more essential for patient care.

Currently, even well-resourced hospitals struggle to keep up with the growing need for the interpretation of genome-sequencing results. One potential solution to this problem of scaling is IBM Watson for Genomics (WfG), a cloud-based service for evidence gathering and genomic analysis, which can be used to analyze large volumes of genome data and obtain evidence-based answers (6, 7). For each patient, WfG reads a data file containing a tumor's genetic variants and evaluates each variant using advanced cognitive analytics against data sources, such as treatment guidelines, peer-reviewed journal articles, and clinical studies. WfG identifies which ones are actionable based on the literature as well as the list of FDA-approved drugs and recruiting clinical trials. The information is presented in a report along with links to the relevant medical literature. The patient's doctor then reviews this information alongside additional clinical evidence to make an informed treatment decision. For this study, only recruiting clinical trials in the United States were used by WfG during the analysis, which might lead to differences in the list of potential therapies for each patient case.

Since April 2013, the National Cancer Center Hospital (NCCH) in Japan has been conducting a clinical genome sequencing project (TOP-GEAR PROJECT). This project was initiated to match patients whose tumors harbor specific molecular aberrations to investigational phase I trials of targeted therapeutics. Preliminary data for this project have already been reported, and in the present study, we used WfG to reanalyze the sequencing results (8). The primary objective of this study was to assess the validity and utility of WfG for analyzing clinical genome sequencing results by comparisons with results obtained by an expert panel composed of multidisciplinary specialists at NCCH.

## Materials and methods

### Patient selection

Patients with solid tumors who were candidates for phase I trials at the NCCH in Japan and who participated in the clinical genome sequencing project (UMIN000011141) between April 2013 and October 2016 were identified. Characteristics and clinical information for the patients were obtained from their medical records. The study is approved by the institutional review boards of the National Cancer Center Hospital (ID: 2012-374). All subjects gave written informed consent in accordance with the Declaration of Helsinki.

### In-house genome sequencing system

In-house genome sequencing was performed with formalin-fixed, paraffin-embedded tissue samples using an original oncogene panel (“NCC oncopanel,” ver. 2.0, containing 90 mutations/amplifications and 10 fusion genes; ver. 3.0, 104 mutations/amplifications, and 16 fusions, Supplementary Table 1). By using SureSelect XT reagent (Agilent Technologies) and a KAPA Hyper Prep kit (KAPA Biosystems, MA, USA), sequencing libraries were prepared, and then the libraries were analyzed on a MiSeq sequencer (Illumina, CA, USA). NGS reads were mapped to the human reference genome by BWA (9) and BWA-SW (10), and gene mutations [single nucleotide variants and short insertions and deletions (indels)], gene amplifications, and gene fusions were detected by using the in-house program. All detected alterations were checked by manual inspection using the Integrative Genomics Viewer, and the list of altered genes was obtained. Single nucleotide polymorphisms (SNPs) were eliminated from the list by using the existing databases. A multidisciplinary team, which included biologists, genome researchers, bioinformaticians, pathologists, and clinicians, annotated each altered gene in the list except for SNPs. For annotation and SNPs elimination, ANNOVAR (11), COSMIC (12), 1000 Genomes (13), ESP6500 (14), Human Genetic Variation Database (15), and in-house Japanese germline SNP data were used. In addition, treatment options, i.e., corresponding molecularly targeted drugs, which included investigational drugs, were reported. The detailed methods were described previously (8, 16).

### Molecular interpretation by WfG

The same list of altered genes was re-analyzed by WfG. The following information was uploaded to WfG for each case: (a) cancer type, (b) a list of variants as a variant calling file (.vcf), (c) gene-level copy number alterations expressed as a log2 ratio of tumor to normal tissues, and (d) fusions represented by gene pairs. Within a few minutes, WfG returned results, which included: (a) variants categorized according to the degree of pathogenicity (benign, likely benign, pathogenic, likely pathogenic, and variant of unknown significance [VUS]); (b) for each actionable pathogenic and likely pathogenic variant, a list of therapeutic options, including FDA-approved drugs and recruiting clinical trials categorized by the level of evidence; (c) resistance information (when applicable). When evaluating gene amplifications, a copy number ≥8 was considered as pathogenic both by the expert panel and WfG. Evidence extracted from peer-reviewed articles, selected databases, and clinical trials used during the analysis were presented in the report in addition to the results. Examples of the input and the output data can be found in the Supplementary Materials (Supplementary Table 2 and Datasheet 1).

This analysis was performed twice using different versions of WfG: version 27 (analysis conducted November 2016) and version 33 (September 2017). WfG became commercially available in October 2016.

### Comparison of results obtained by the expert panel and WfG

First, analytical results, including proposed pathogenic gene alterations and targetable pathways, obtained by the expert panel and WfG (ver. 27) were compared. By comparing the results obtained using WfG ver. 27 and ver. 33, the temporal progress of the WfG system was evaluated, with a focus on previously discrepant cases. For comparison to the expert panel, the WfG results were grouped as follows; pathogenic and likely pathogenic mutations were categorized as pathogenic alterations, whereas VUSs and likely benign/benign mutations were categorized as non-pathogenic alterations.

### Statistics

The 95% confidence interval [CI] for each rate was calculated using R Version 3.4.2 (R Foundation for Statistical Computing, Vienna, Austria). The Cohen's kappa statistic was used to measure agreement between the results of the expert panel and WfG.

## Results

### Top-gear project

**Figure 1:**
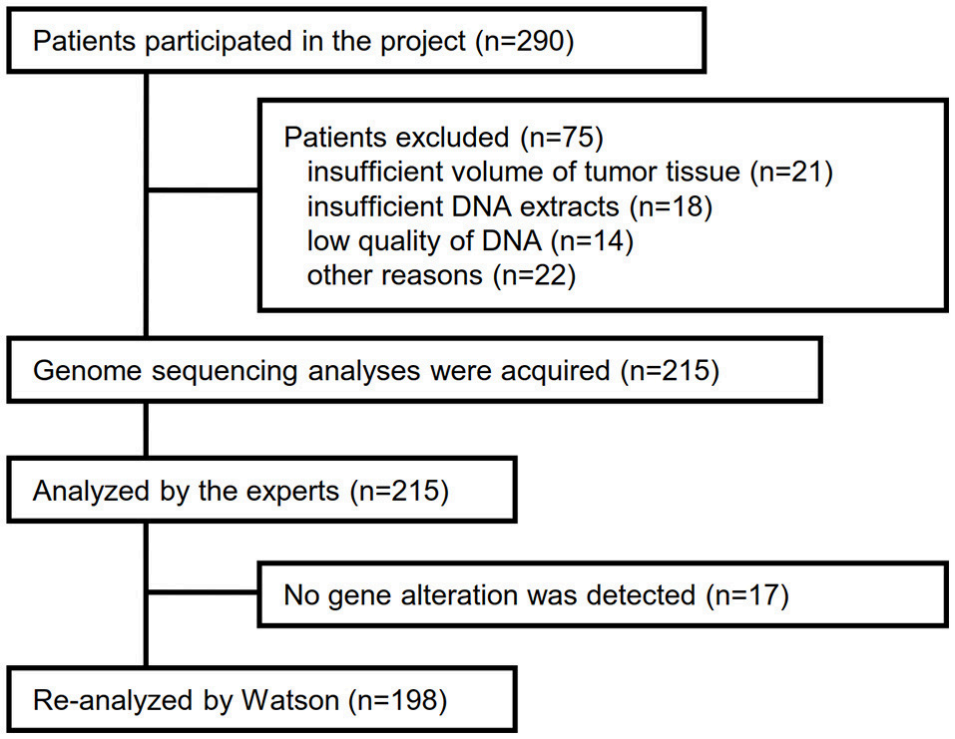
Flow diagram for selection of cancer patients in this analysis.

**Figure 2:**
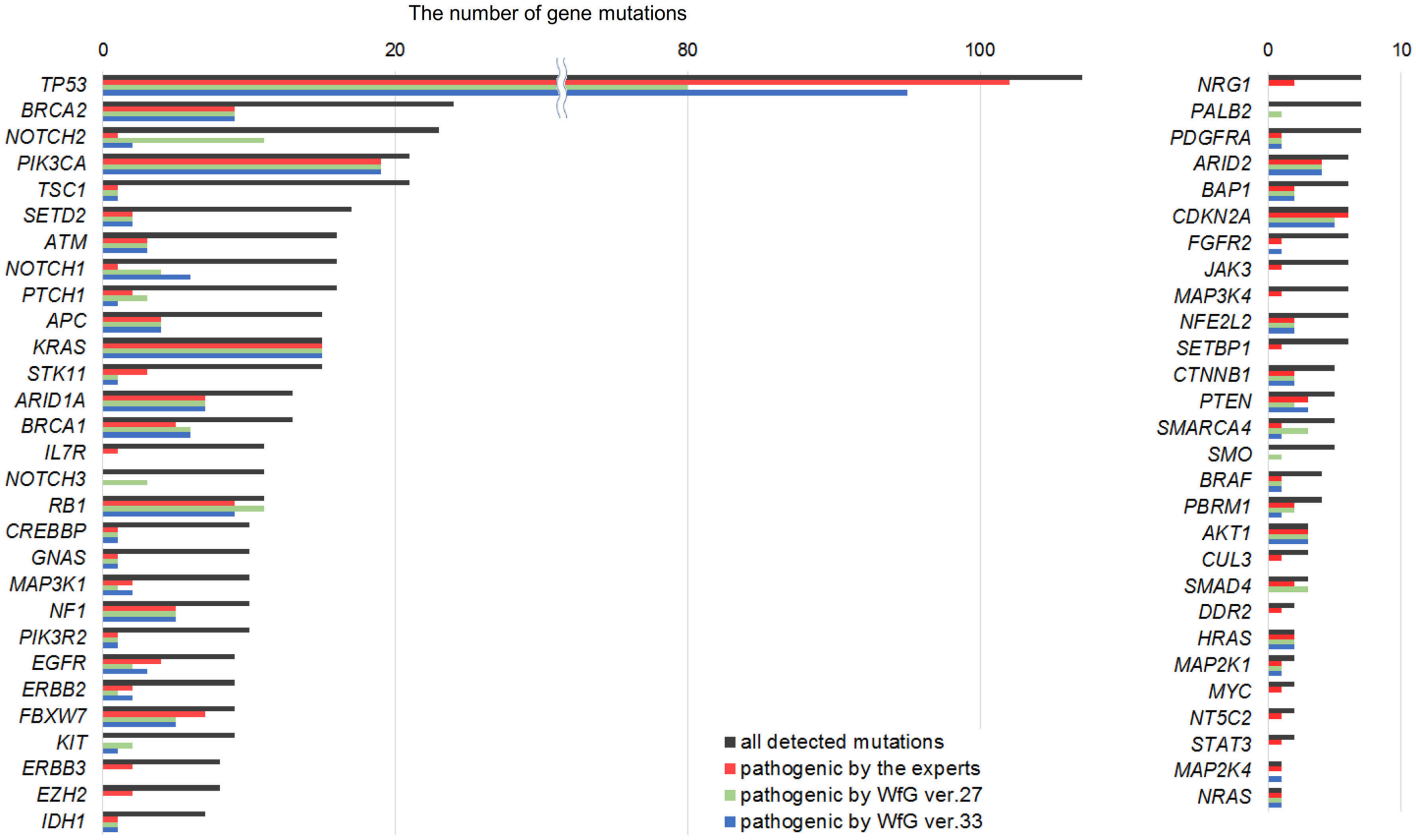
List of all gene mutations and pathogenic mutations detected by the expert panel, WfG ver. 27 and WfG ver.33. The number of all gene mutations detected (excluding SNPs), the number of pathogenic mutations identified by experts, the number of pathogenic mutations identified by WfG ver. 27, and the number of pathogenic mutations identified by WfG ver. 33 are shown. Genes without detected pathogenic mutations are not shown.

**Figure 3:**
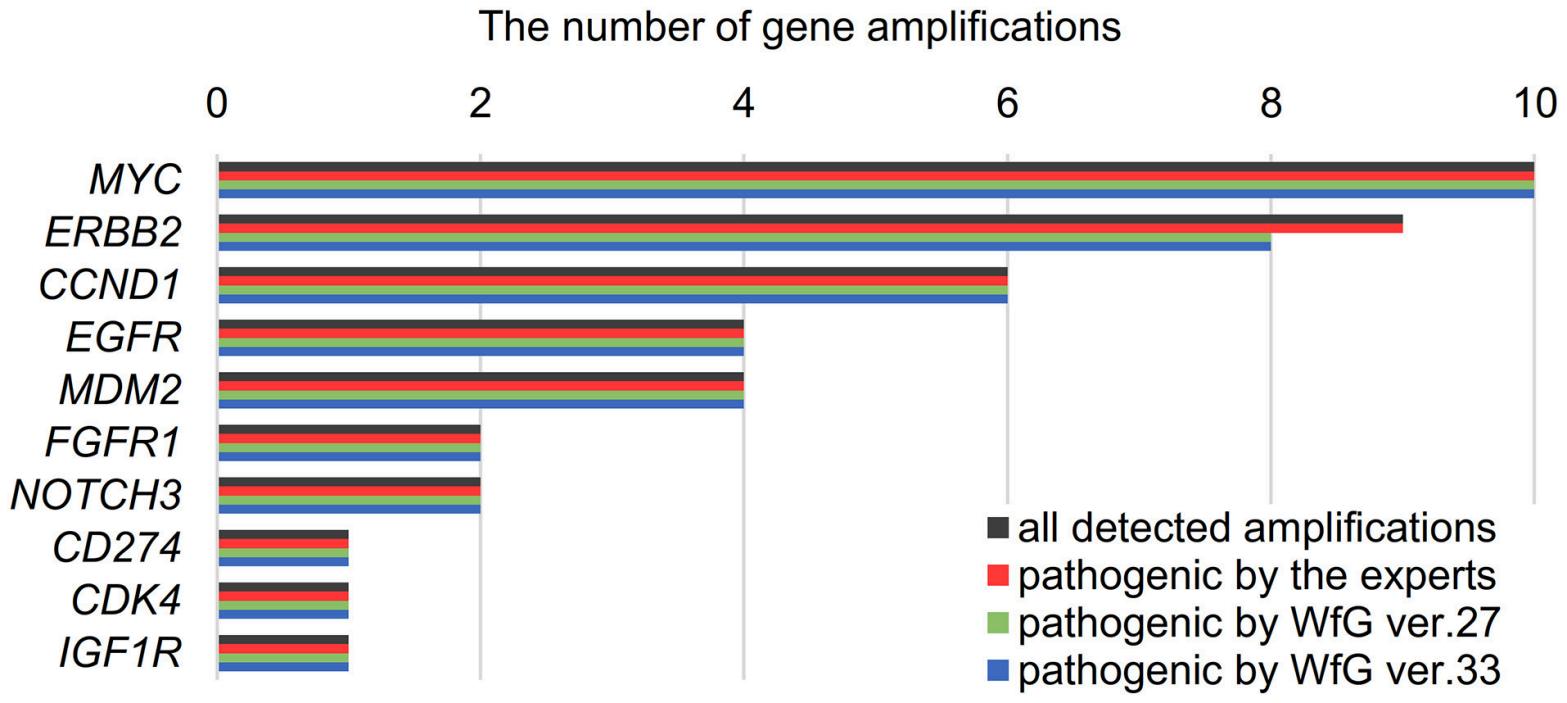
List of all gene amplifications and pathogenic amplifications detected by the expert panel, WfG ver. 27 and WfG ver.33. The number of all gene amplifications detected, the number of pathogenic amplifications identified by experts, the number of pathogenic amplifications identified by WfG ver. 27, and the number of pathogenic amplifications identified by WfG ver. 33 are shown.

Between April 2013 and October 2016, the clinical genomic sequencing project registered 290 patients with cancer and sequencing data was acquired from 215 patients (Figure 1). No gene alterations were detected in 17 patients; therefore, 198 patients were included in this study. Patient characteristics are summarized in Table 1. Breast cancer (*n* = 40, 20.2%) was the most frequent diagnosis in this analysis, followed by gastric cancer (*n* = 31, 15.7%), and lung cancer (*n* = 30, 15.2%). The list of altered genes, excluding SNPs, included 785 mutations, 40 amplifications, and 22 fusions (Supplementary Tables 3–5). The genes *TP53* (*n* = 107), *BRCA2* (*n* = 24), and *NOTCH2* (*n* = 23) had the highest frequency of SNVs. *MYC* (*n* = 10) was the most frequently detected gene amplification, followed by *ERBB2* (*n* = 9) and *CCND1* (*n* = 6). From these gene alterations, 257 mutations, 40 amplifications, and 4 fusions (*KIF5B-RET, CD74- ROS1, CLTC-ALK*, and *GBA3*-*ALK*) were judged as pathogenic by the expert panel (Supplementary Table 6). The proportion of pathogenic mutations and amplifications detected for each gene is summarized in Figures 2, 3.

**Table 1:** Characteristics of patients.

**Characteristic**	**Subgroup**	**Value**
Age, mean (range)	Years	58 (21–78)
Sex, *n* (%)	Male	71 (35.9%)
Primary site, *n* (%)	Breast	40 (20.2)
	Gastric	31 (15.7)
	Lung	30 (15.2)
	Ovary	22 (11.1)
	Sarcoma	13 (6.6)
	Neuroendocrine	10 (6.1)
	Bile duct	9 (4.5)
	Thymic	7 (3.1)
	Cervical	5 (2.5)
	Urine body	5 (2.5)
	Colon	3 (1.5)
	Pancreas	3 (1.5)
Gene mutations, *n*	*TP53*	107
	*BRCA2*	24
	*NOTCH2*	23
	*PIK3CA*	21
	*TSC1*	21
	*RET*	19
	*ROS1*	19
	*SETD2*	17
	*ATM*	16
	*NOTCH1*	16
	*PTCH1*	16
	*APC*	15
	*KRAS*	15
	*STK11*	15
	*ALK*	14
	*ABL1*	13
	*ARID1A*	13
	*BRCA1*	13
	others	388
gene Amplifications, copy number	*MYC*	10
	*ERBB2*	9
	*CCND1*	6
	*EGFR*	4
	*MDM2*	4
	*FGFR1*	2
	*NOTCH3*	2
	*CD274*	1
	*CDK4*	1
	*IGF1R*	1

### Gene mutations analyzed in WfG ver. 27 and concordance with the expert panel

**Figure 4:**
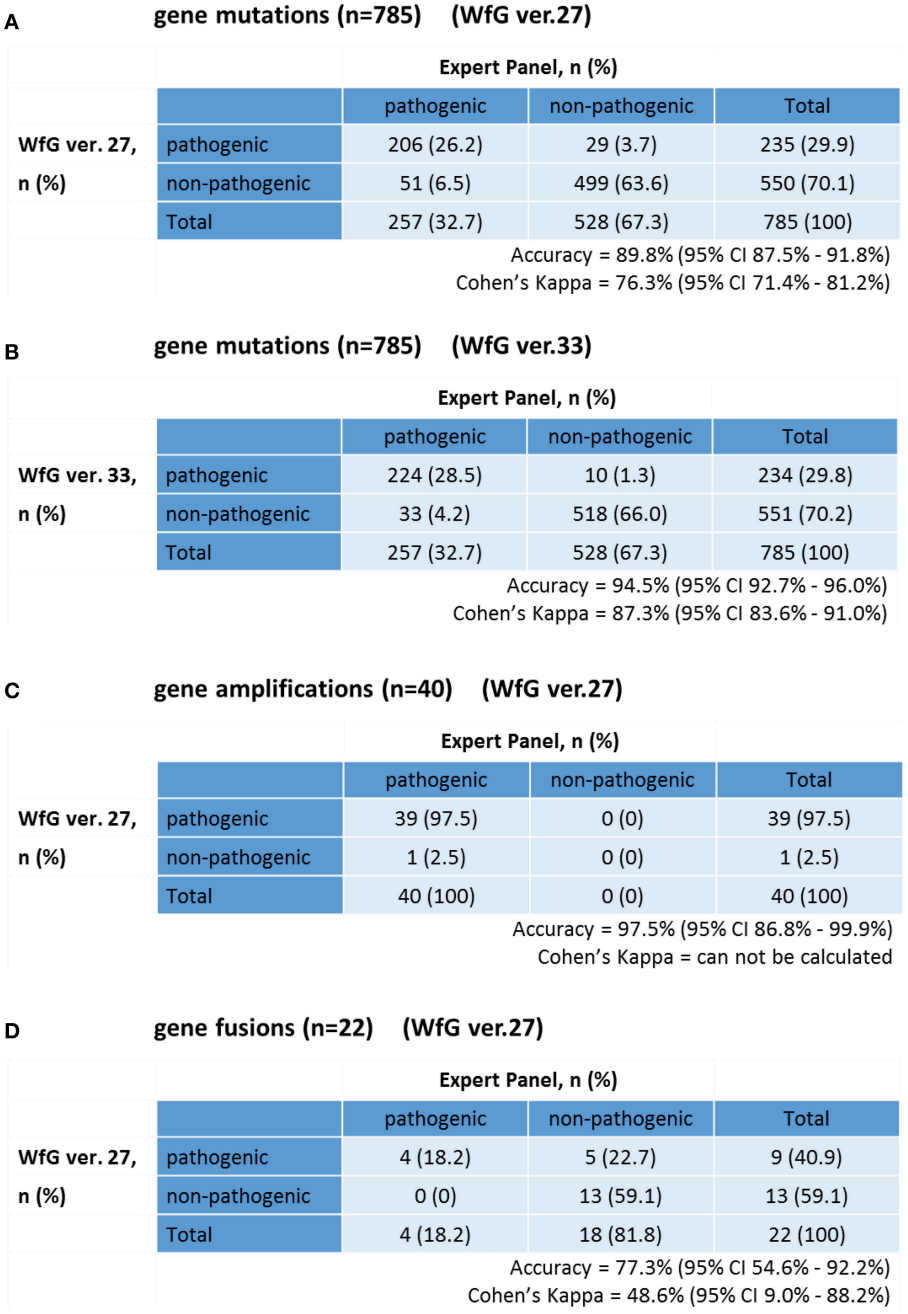
Concordance of pathogenic evaluations of gene mutations, gene amplifications, and gene fusions between the expert panel and WfG **(A)**. Concordance of pathogenic gene mutation detection using WfG ver. 27 **(B)**. Concordance of pathogenic gene mutation detection using WfG ver. 33 **(C)**. Concordance of pathogenic gene amplification detection using WfG ver. 27 **(D)**.

The same list of altered genes, excluding SNPs, was analyzed using WfG ver. 27, and 235 of 785 mutations, 39 of 40 amplifications, and 9 of 22 fusions were identified as pathogenic (Figures 2, 3, and Supplementary Table 7). We compared the results obtained by WfG ver. 27 and the expert panel. Of 785 gene mutations, 206 mutations (26.2%) were identified as pathogenic by both methods, and 499 mutations (63.6%) were non-pathogenic by both methods (Figure 4A). The total number of concordant decisions for mutation pathogenesis was 705 out of 785 (89.8%; 95% CI, 87.5–91.8%). Of the 80 mutations identified as pathogenic by either method, 51 mutations (6.5%) were identified only by the expert panel, and the other 29 mutations (3.7%) were identified only by WfG.

**Figure 5:**
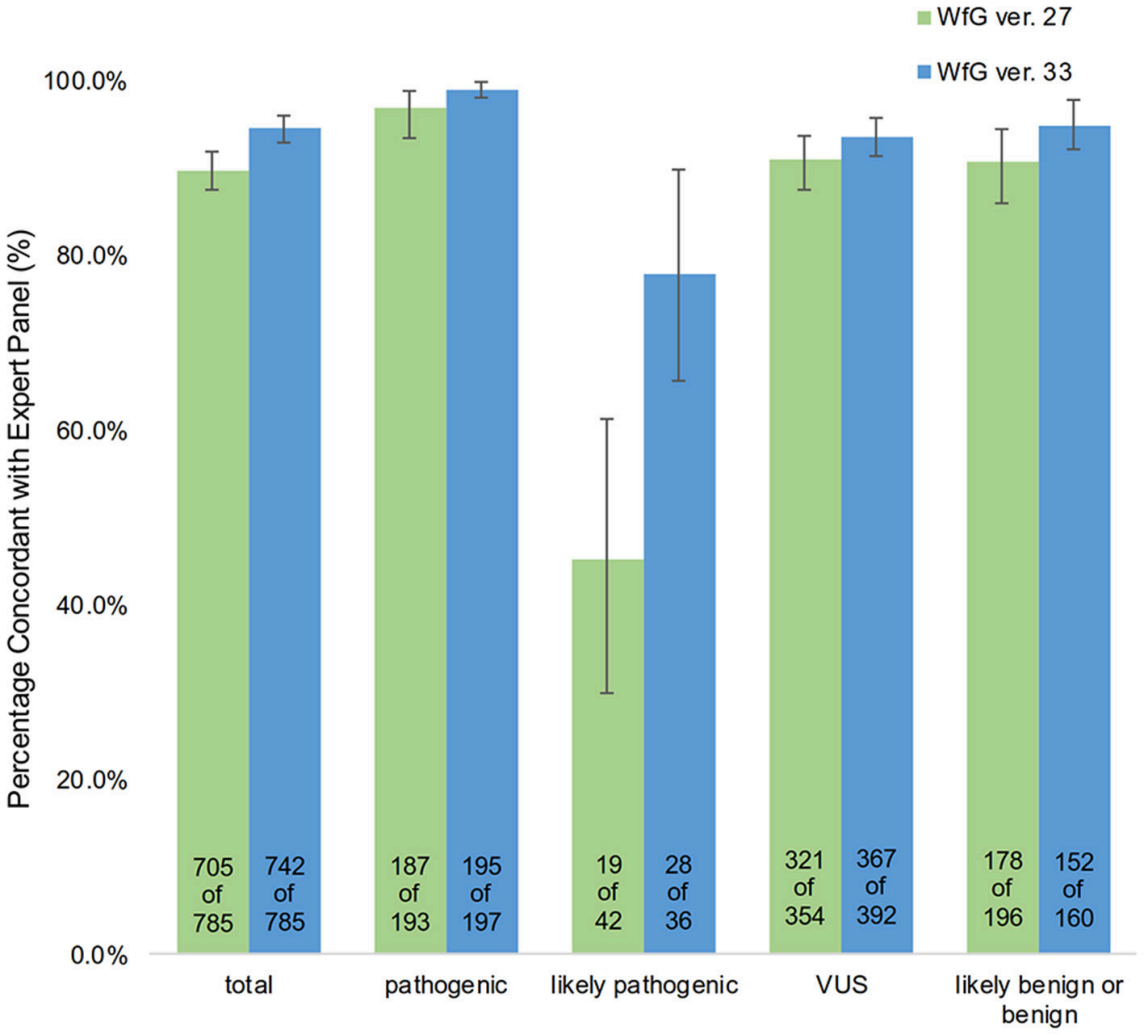
Concordance of pathogenic evaluations of gene mutations between the expert panel and WfG (breakdown of concordant and discordant cases by WfG pathogenic subgroup).

The degree of gene pathogenicity was stratified into five groups by WfG ver. 27, and concordance was further analyzed based on this classification (Figure 5). Of the 785 total mutations, 193 mutations were categorized as pathogenic, 42 mutations as likely pathogenic, 354 mutations as VUS, and 196 mutations as likely benign or benign. In the pathogenic subgroup, 187 out of 193 (96.9%; 95% CI, 93.3–98.9%) mutations were also identified as pathogenic mutations by the expert panel. In the likely pathogenic subgroup, 19 out of 42 (45.2%, 95% CI, 29.8–61.3%) mutations were identified as pathogenic by the panel. In the VUS subgroup, 321 out of 354 (90.7%, 95% CI, 87.2–93.5%) mutations were determined to be non-pathogenic by the panel and in the likely benign/benign subgroup, 178 out of 196 mutations (90.8%; 95% CI, 85.9–94.5%) were determined to be non-pathogenic by the panel.

### Causes of discrepancies in the evaluation of pathogenicity between the expert panel and WfG ver. 27, and reanalysis by WfG ver. 33

Eighty unmatched mutations were discussed between investigators at the NCCH and at IBM, and the reasons for the discrepancies were identified. Detailed data for genes with unmatched mutations are summarized in Table 2. There was a high possibility that 24 out of 80 unmatched mutations were likely false positive assessments by WfG ver. 27, and 22 unmatched mutations were likely false negative assessments by WfG. For example, EGFR G719C is a well-known actionable mutation in patients with lung adenocarcinoma (17, 18), and patients with FGFR W290C mutation-positive tumor were reported to be sensitive to FGFR inhibitors (19), but these mutations were determined as VUSs by WfG ver.27 (false negative examples). Assessments for 14 TP53 mutations (R156P, Y205C, H214R, etc.) determined as VUSs by WfG ver.27 (false negative examples) and those for 14 NOTCH2-3 mutations determined as likely pathogenic (false positives examples) were reversed after discussion. Pathogenic assessments about TP53 or NOTCH families by WfG ver.27 needed improvement. Conversely, two decisions by the expert panel were reversed after further discussion. These insufficient assessments are summarized in Supplementary Table 8. In addition, the expert panel identified five VUSs as pathogenic mutations out of abundant caution. The expert panel found 14 mutations to be pathogenic based only on the observation of one or more reports in the COSMIC (12) database of their presence in tumors. The lower threshold of pathogenicity held by the expert panel when evaluating VUSs or using COSMIC may explain these 19 discrepant evaluations. Opinions regarding the remaining 13 mutations were divided between investigators because different references were used to determine the degree of pathogenicity, and the pathogenicity of the mutations was not always sufficiently established.

**Table 2:** The number and the proportion of unmatched mutations between the expert panel and WfG (in genes with ≥2 pathogenic mutations detected by both analyses).

**Gene names**	**Total number of mutations**	**Number of discordant results using WfG ver. 27, (*n*, %)**	**Number of discordant results using WfG ver. 33, (*n*, %)**
*NOTCH2*	23	12 (52.2)	3 (13.0)
*SMARCA4*	5	2 (40.0)	0 (0.0)
*SMAD4*	3	1 (33.3)	2 (66.7)
*MAP3K1*	10	3 (30.0)	0 (0.0)
*NRG1*	7	2 (28.6)	2 (28.6)
*NOTCH3*	11	3 (27.3)	0 (0.0)
*ERBB3*	8	2 (25.0)	2 (25.0)
*EZH2*	8	2 (25.0)	2 (25.0)
*EGFR*	9	2 (22.2)	1 (11.1)
*FBXW7*	9	2 (22.2)	2 (22.2)
*KIT*	9	2 (22.2)	1 (11.1)
*TP53*	107	22 (20.6)	8 (7.5)
*NOTCH1*	16	3 (18.8)	5 (31.3)
*RB1*	11	2 (18.2)	0 (0.0)
*STK11*	15	2 (13.3)	2 (13.3)
*PTCH1*	16	1 (6.3)	1 (6.3)
Others	518	17 (3.3)	12 (2.3)
Total	785	80 (10.2)	43 (5.5)

**Figure 6:**
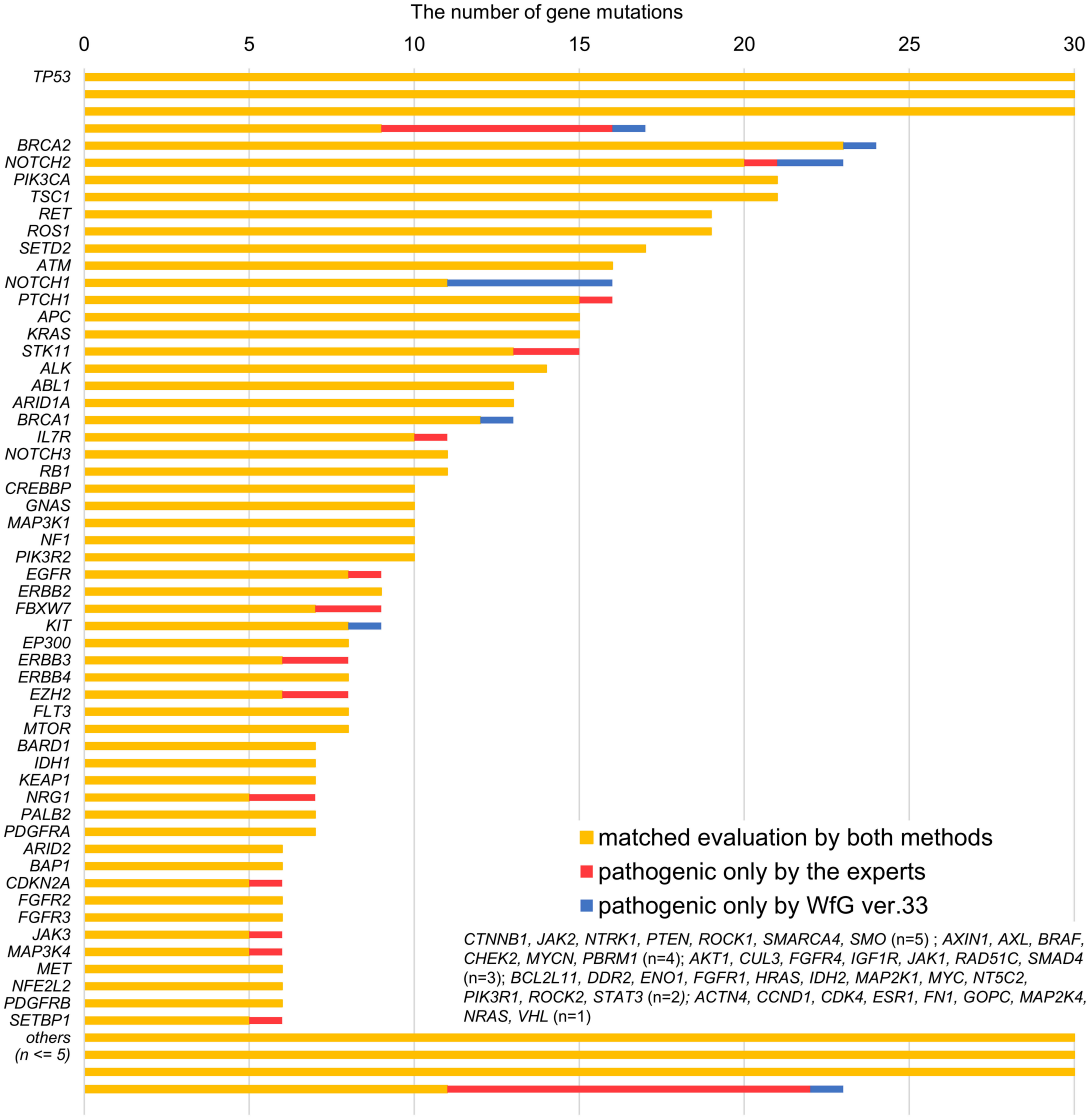
Most recent concordance results for gene mutation pathogenicity between the expert panel and WfG ver. 33 by gene.

After reanalysis using WfG ver. 33, 46 of the 80 unmatched mutations became concordant with the expert panel results (Figure 4B). The analysis using ver. 27 resulted in many disagreements in *TP53* and *NOTCH2-3*. Ver. 33 was improved with respect to *TP53* and *NOTCH2-3*, and the number of disagreements was substantially reduced. *EGFR* G719C, *FGFR2* W290C, and *PTEN* P246L (20) were updated from VUS to pathogenic. However, nine new unmatched mutations were observed. The total number of concordant decisions increased from 705 mutations to 742 mutations (94.5%, 95% CI, 92.7–96.0%). The number of concordant assignments with respect to mutation pathogenicity after reanalysis is broken down by gene in Figure 6. The proportion of matched cases increased after reanalysis for all subgroups (Figure 5).

### Gene amplifications analyzed by WfG ver. 27

The concordance of pathogenic evaluations of gene amplifications between the expert panel and WfG ver. 27 was 97.5% (39/40; 95% CI, 86.8–99.9%; Figure 4C). The concordance rate for gene amplifications was high; however, one case of *ERBB2* amplification was proposed as pathogenic only by the expert panel. Copy number variation in this gene was found to be more than eight copies; therefore, the authors believe this was a false negative result by WfG. The results of the comparison about gene gene amplifications and fusions were not reported because the same results were obtained after the reanalysis by ver.33.

### Fusions analyzed in WfG ver. 27

Four fusions (*KIF5B-RET, CD74- ROS1, CLTC-ALK*, and *GBA3*-*ALK*) were determined as valid and pathogenic by both the expert panel and WfG ver. 27 (Figure 4D). Three of these fusions are well-known in patients with lung cancer. The fourth, *GBA3*-*ALK*, has not been reported previously, and a functional analysis of this fusion has not been conducted; therefore, the role of this fusion in tumorigenesis is not clear. WfG reported an additional five fusions (Supplementary Table 7) as pathogenic. *BRAF-SULT4A1* and its reciprocal fusion *SULT4A1-BRAF* were evaluated as pathogenic; *BRAF* fusions, such as *KIAA1549-BRAF*, have been reported in astrocytoma, (21) supporting the conclusion that this fusion is likely pathogenic. *FIP1L1-PDGFRA* is a recurrent fusion in chronic eosinophilic leukemia (22). Although it has not been detected in solid tumors, this result is plausible. However, due to low read frequency, the authors believe that these were sequencing artifacts. Overall concordance was 77.3% (95% CI, 54.6–92.2%) for fusions.

### Targeted drugs

**Figure 7:**
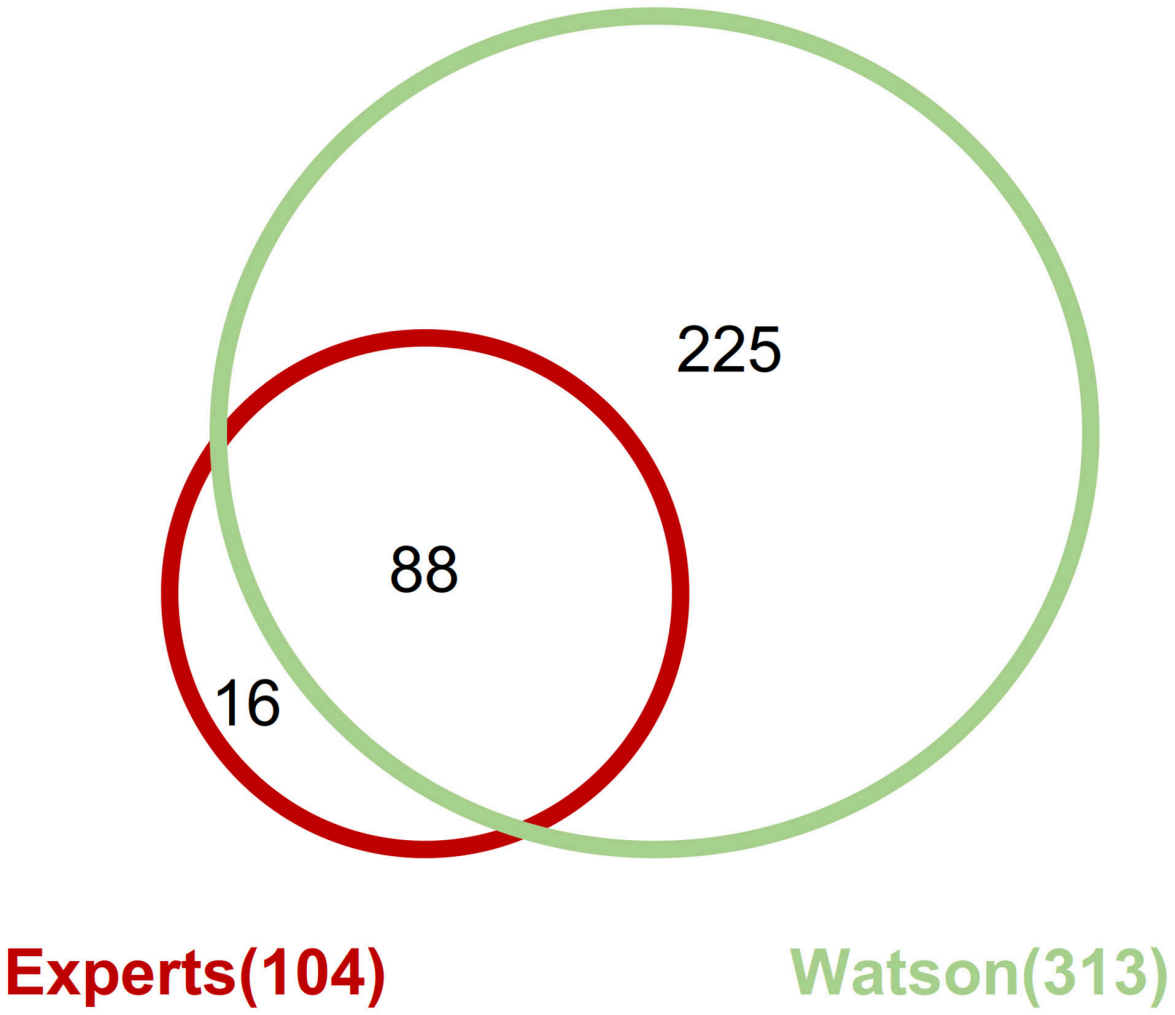
Concordance of proposed targeted drugs between the experts and Watson System against gene alterations determined as pathogenic by both method including 206 mutations, 39 amplifications, and 4 fusions.

Across all the detected pathogenic gene alterations in this analysis, the expert panel proposed targeted drugs against 113 pathways including 111 gene alterations; WfG ver. 27 proposed drugs targeting 322 pathways and 227 alterations. When drugs targeting the same signaling pathway were proposed for a gene alteration, they were combined into the same pathway-targeted drug for the analysis. For example, drugs targeting *RAS, RAF, MEK*, and *ERK* were grouped into a single *RAS-RAF-MEK-ERK* signaling pathway-targeted drug. When focusing on the pathogenic gene alterations detected by both the expert panel and WfG ver. 27 (including 206 mutations, 39 amplifications, and 4 fusions), 104 pathways with 102 gene alterations and 313 pathways with 218 gene alterations were proposed as targetable by the expert panel and WfG ver. 27, respectively (Supplementary Table 9). Thus, WfG proposed about twice as many targetable gene alterations and three times as many targeted drugs. Of the 104 pathways with 102 gene alterations for which targeted drugs were proposed by the expert panel, 88 (84.6%) of the same pathway-targeted drugs were also proposed by WfG (Figure 7). Thus, WfG covered most targeted therapies proposed by the expert panel and offered additional therapeutic options, including investigational drugs in recruiting trials

## Discussion

This is the first detailed analysis of WfG results in a temporal progression. WfG exhibited a high level of concordance with our expert panel for the identification of pathogenic gene mutations and amplifications. The update process of WfG was not related to this study and had been performed independently. The difference in performance by WfG from ver. 27 to ver. 33 is primarily due to three factors. First, WfG knowledge corpus was expanded through ingestion of new articles and the addition of several databases, including OncoKB. Second, a new, more accurate functional annotation tool was incorporated into the software. Third, the system continued to learn through training on additional cases from other clients leading to an overall improvement in the concordance rate (23).

The concordance rate between WfG ver. 27 and the panel for gene mutations was 89.8%. After reanalysis using WfG ver. 33, there was a 4.7% increase in concordance for gene mutations. Most of the remaining discrepancies in the assigned pathogenicity of gene mutations can be explained by a lack of evidence supporting the function of genes in cancer. Large datasets for genome alterations in cancer are available from several genome databases, but the pathogenicity has not been sufficiently established for many gene alterations, and the degree of pathogenicity is often unclear. Evaluations of non-synonymous mutations and in-frame indels in tumor suppressor genes can be quite challenging owing to insufficient information from the literature. The increase in concordance from ver. 27 to ver. 33 demonstrates the cognitive capabilities of WfG, which continues to learn as more cases are interpreted.

Gene alterations were more likely to be identified as pathogenic by the expert panel. In daily clinical practice, it is important to offer additional treatment options for patients with cancer. Therefore, the expert panel may have a lower threshold for determining the degree of pathogenicity. In contrast, WfG offered targeted therapies against about twice as many gene alterations compared with the expert panel. Moreover, WfG covered about 84.5% of targeted therapies proposed by the expert panel. The proposal of investigational drugs as a therapeutic option is usually a difficult challenge. The expert panel required stricter evidence when selecting targeted therapeutic options, whereas WfG is designed to be more inclusive of potential therapies, providing the level of evidence for each potential therapy and enabling the clinician to make the final treatment decision.

At present, WfG is useful for a clinician at a general hospital although an additional survey of evidence by a clinician is required when evaluating fusions. WfG can also offer additional and beneficial opinions including investigational drugs even for a clinician at a cancer specialty hospital. In the near future, genome sequencing of patients' tumors and molecularly targeted therapies may become more common, and a shortage of specialists is expected. Additionally, the amount of data collected per patient is expected to increase. For this study, we conducted targeted sequencing; however, analyses of exome-sequencing data or whole-genome sequencing data will become necessary, further increasing the burden on specialists. The use of cognitive technologies, such as WfG, is a promising option for overcoming this scaling problem.

The usage of WfG has some limitations. WfG interpreted.vcf files. The processes of mapping reads from raw sequencing data obtained from MiSeq (fastq) and removing mapping errors were performed by bioinformaticians at NCCH. Our study also had some limitations. In particular, the number of evaluated amplifications and fusions was low for a comprehensive evaluation of accuracy. Additionally, although the validity and utility of WfG were judged based on its agreement with the opinions of experts at our hospital, the pathogenicity of many gene alterations in tumors has not been established, and interpretations were not always correct or clear. The validity and utility in clinical trials, including basket studies of targeted therapies, should be evaluated by accumulating additional clinical data, such as tumor responses or non-responses to matched targeted drugs proposed by WfG. Moreover, the opinions of the expert panel at our institution were based on data obtained from many databases available at the time of the analysis. Thus, it is necessary to analyze differences in results as databases are updated.

In conclusion, WfG showed comparable analytical results for clinical genome sequencing to those of a multidisciplinary team at a cancer specialty hospital. A few discrepancies in gene mutations and amplifications remained after reanalysis, and there was room for improvement in the analysis of gene fusions. WfG development continues, and it demonstrated a significant improvement in mutation assignment from ver. 27 and 33. WfG may be useful in cases where large amounts of genomic data is available, such as whole exon sequences, and in institutions with an insufficient number of experts in gene analyses. However, additional evaluation of Watson for Genomics in clinical trials is necessary to further validate its assessments and drug recommendations.

## Author contributions

KI, SK, and NY contributed conception and design of the study. KI and SK organized the database. KI performed the statistical analysis. KI and SK wrote the first draft of the manuscript. All authors contributed to manuscript revision, read and accepted the final version of the manuscript.

### Conflict of interest statement

YF has provided consulting for Ono Pharmaceutical and Bristol-Myers Squibb Japan; participated in speakers' Bureau for Ono Pharmaceutical, Bristol-Myers Squibb Japan, MSD, and Taiho Pharmaceutical; and received research funding from AstraZeneca, Chugai Pharmaceutical, Daiichi Sankyo, Eisai, Lilly Japan, Novartis, Bristol-Myers Squibb Japan, MSD, Merck Serono, Abbvie, and Incyte. NY has provided consulting for Eisai, Takeda, Otsuka Pharmaceutical, and OncoTherapy Science; participated in speakers' Bureau for Bristol-Myers Squibb Japan, Pfizer, AstraZeneca, Lilly Japan, Ono Pharmaceutical, and Chugai Pharmaceutical; and received research funding from Chugai Pharmaceutical, Taiho Pharmaceutical, Eisai, Quintiles, Astellas Pharma, Novartis, Daiichi Sankyo, Boehringer Ingelheim, Takeda, Kyowa Hakko Kirin, Bayer, Pfizer, and Bristol-Myers Squibb Japan, Ono Pharmaceutical, and Bayer. TKoy, RT, JX, CH, VM, and LP, are employees of and own stock in IBM. The remaining authors declare that the research was conducted in the absence of any commercial or financial relationships that could be construed as a potential conflict of interest.
