# Supplementary material for: Evaluating Clinical Genome Sequence Analysis by Watson for Genomics
Source: Front Med (Lausanne). 2018 Nov 9;5:305. doi: 10.3389/fmed.2018.00305 (PMC6237914; doi:10.3389/fmed.2018.00305)
Supplement: Supplementary file 1 [file Table_1.DOCX]

**Supplementary Table S1.** Gene list of NCC oncopanel ver.2 and ver.3 used for targeted sequencing.

| Gene list of NCC oncopanel ver. 2 | | |  |
| --- | --- | --- | --- |
| Genes for mutations and amplifications | | | Genes for fusions |
| *ABL1* | *FGFR2* | *PALB2* | *AKT3* |
| *AKT1* | *FGFR3* | *PBRM1* | *ALK* |
| *AKT2* | *FGFR4* | *PDGFRA* | *BRAF* |
| *AKT3* | *FLT3* | *PDGFRB* | *FGFR2* |
| *ALK* | *HRAS* | *PIK3CA* | *FGFR3* |
| *APC* | *IDH1* | *PIK3R1* | *NOTCH1* |
| *ARID1A* | *IDH2* | *PTCH1* | *NRG1* |
| *ARID2* | *IGF1R* | *PTEN* | *RAF1* |
| *ATM* | *IGF2* | *RAC1* | *RET* |
| *AXIN1* | *IL7R* | *RAC2* | *ROS1* |
| *BAP1* | *JAK1* | *RAD51C* |  |
| *BARD1* | *JAK2* | *RAF1* |  |
| *BIM* | *JAK3* | *RB1* |  |
| *BRAF* | *KEAP1* | *RET* |  |
| *BRCA1* | *KIT* | *ROS1* |  |
| *BRCA2* | *KRAS* | *SETD2* |  |
| *CCND1* | *MAP2K1* | *SMAD4* |  |
| *CDK4* | *MAP2K4* | *SMARCA4* |  |
| *CDKN2A* | *MAP3K1* | *SMO* |  |
| *CHEK2* | *MAP3K4* | *STAT3* |  |
| *CREBBP* | *MDM2* | *STK11* |  |
| *CTNNB1* | *MET* | *TP53* |  |
| *CUL3* | *MTOR* | *TSC1* |  |
| *DDR2* | *MYC* | *VHL* |  |
| *EGFR* | *MYCN* |  |  |
| *ENO1* | *NF1* |  |  |
| *EP300* | *NFE2L2* |  |  |
| *ERBB2* | *NOTCH1* |  |  |
| *ERBB3* | *NOTCH2* |  |  |
| *ERBB4* | *NOTCH3* |  |  |
| *EZH2* | *NRAS* |  |  |
| *FBXW7* | *NRG1* |  |  |
| *FGFR1* | *NT5C2* |  |  |

| Gene list of NCC oncopanel ver. 3 | |  |  |
| --- | --- | --- | --- |
| Genes for mutations and amplifications | | | Genes for fusions |
| *ABL1* | *EZH2* | *NRAS* | *AKT3* |
| *ACTN4* | *FBXW7* | *NRG1* | *ALK* |
| *AKT1* | *FGFR1* | *NTRK1* | *AXL* |
| *AKT2* | *FGFR2* | *NT5C2* | *BRAF* |
| *AKT3* | *FGFR3* | *PALB2* | *EGFR* |
| *ALK* | *FGFR4* | *PBRM1* | *ERBB4* |
| *APC* | *FLT3* | *PDGFRA* | *FGFR2* |
| *ARID1A* | *GNAS* | *PDGFRB* | *FGFR3* |
| *ARID2* | *HRAS* | *PIK3CA* | *NOTCH1* |
| *ATM* | *IDH1* | *PIK3R1* | *NRG1* |
| *AXIN1* | *IDH2* | *PIK3R2* | *NTRK1* |
| *AXL* | *IGF1R* | *PRKCl* | *RAF1* |
| *BAP1* | *IGF2* | *PTCH1* | *RET* |
| *BARD1* | *IL7R* | *PTEN* | *ROS1* |
| *BIM* | *JAK1* | *RAC1* |  |
| *BRAF* | *JAK2* | *RAC2* |  |
| *BRCA1* | *JAK3* | *RAD51C* |  |
| *BRCA2* | *KEAP1* | *RAF1* |  |
| *CCND1* | *KIT* | *RB1* |  |
| *CD274* | *KRAS* | *RET* |  |
| *CDK4* | *MAP2K1* | *RHOA* |  |
| *CDKN2A* | *MAP2K4* | *ROCK1* |  |
| *CHEK2* | *MAP3K1* | *ROCK2* |  |
| *CRKL* | *MAP3K4* | *ROS1* |  |
| *CREBBP* | *MDM2* | *SETBP1* |  |
| *CTNNB1* | *MDM4* | *SETD2* |  |
| *CUL3* | *MET* | *SMAD4* |  |
| *DDR2* | *MTOR* | *SMARCA4* |  |
| *EGFR* | *MYC* | *SMO* |  |
| *ENO1* | *MYCN* | *STAT3* |  |
| *EP300* | *NF1* | *STK11* |  |
| *ERBB2* | *NFE2L2* | *TP53* |  |
| *ERBB3* | *NOTCH1* | *TSC1* |  |
| *ERBB4* | *NOTCH2* | *VHL* |  |
| *ESR1/ER* | *NOTCH3* |  |  |
