# Supplementary material for: Evaluating Clinical Genome Sequence Analysis by Watson for Genomics
Source: Front Med (Lausanne). 2018 Nov 9;5:305. doi: 10.3389/fmed.2018.00305 (PMC6237914; doi:10.3389/fmed.2018.00305)
Supplement: Supplementary file 6 [file Table_6.docx]

**Supplementary File S6.** Pathogenic genes detected by the expert panel in TOP-GEAR PROJECT

| **Mutations, gene (N)** | **Amplifications, gene (N)** | **Fusions, gene (N)** |
| --- | --- | --- |
| *TP53* (102) | *MYC* (10) | *KIF5B-RET* |
| *PIK3CA* (19) | *ERBB2* (9) | *CD74-ROS1* |
| *KRAS* (15) | *CCND1* (6) | *CLTC-ALK* |
| *BRCA2* (9) | *EGFR* (4) | *GBA3- ALK* |
| *RB1* (9) | *MDM2* (4) |  |
| *ARID1A* (7) | *FGFR1* (2) |  |
| *FBXW7* (7) | *NOTCH3* (2) |  |
| *CDKN2A* (6) | *CD274* (1) |  |
| *BRCA1* (5) | *CDK4* (1) |  |
| *NF1* (5) | *IGF1R* (1) |  |
| *APC* (4) |  |  |
| *ARID2* (4) |  |  |
| *EGFR* (4) |  |  |
| others (61) |  |  |
