# Supplementary material for: Evaluating Clinical Genome Sequence Analysis by Watson for Genomics
Source: Front Med (Lausanne). 2018 Nov 9;5:305. doi: 10.3389/fmed.2018.00305 (PMC6237914; doi:10.3389/fmed.2018.00305)
Supplement: Supplementary file 8 [file Table_8.DOCX]

**Supplementary File S8.** Change of the pathogenic evaluations about gene alterations after discrepant analysis.

| Experts | likely false positive assessment: *IL7R* (n=1), *SETBP1* (n=1) |
| --- | --- |
| Watson ver.27 | likely false negative assessment: *TP53* (n=14), *EGFR* (n=2), *MAP3K1* (n=2), *FBXW7* (n=1), *FGFR2* (n=1), *MAP3K4* (n=1), *PTEN* (n=1)  likely false positive assessment: *NOTCH2* (n=11), *NOTCH3* (n=3), *RB1* (n=2), *SMARCA4* (n=2), *KIT* (n=1), *MAP3K1* (N=1), *PALB2* (n=1), *PTCH1* (n=1), *SMAD4* (n=1), *SMO* (n=1) |
