# Supplementary material for: Evaluating Clinical Genome Sequence Analysis by Watson for Genomics
Source: Front Med (Lausanne). 2018 Nov 9;5:305. doi: 10.3389/fmed.2018.00305 (PMC6237914; doi:10.3389/fmed.2018.00305)
Supplement: Supplementary file 9 [file Table_9.docx]

**Supplementary File S9.** Proposed targeted therapies by the experts and Watson system against gene alterations determined as pathogenic by both methods Including 206 mutations, 39 amplifications, and 4 fusions.

| **Targeted genes,**  **gene (n)** | **Proposed drugs by experts,**  **class (n)** | **Proposed drugs by Watson,**  **class (n)** |
| --- | --- | --- |
| Mutations | | |
| *AKT1* (3) | AKT inhibitor (3) | AKT inhibitor (3): med |
| *APC* (4) |  | CTNNB1 inhibitor (3): low |
| *ARID1A* (7) |  | PARP inhibitor (7): med – high |
| *ARID2* (4) |  | PARP inhibitor (2): med-high |
| *ATM* (3) | PARP inhibitor (3) | PARP inhibitor (3): med  ATR inhibitor (3): low |
| *BAP1* (2) | PARP inhibitor (2)  EZH2 inhibitor (1) |  |
| *BRAF* (1) | BRAF inhibitor (1) | BRAF inhibitor (1): high  MAP2K2/MAP2K1 inhibitor (1): high |
| *BRCA1* (5) | PARP inhibitor (5) | PARP inhibitor (5): high |
| *BRCA2* (8) | PARP inhibitor (8) | PARP inhibitor (8): high |
| *CDKN2A* (5) | CDK4/6 inhibitor (4) | CDK4/6 inhibitor (5): med |
| *CREBBP* (1) |  | HDAC inhibitor (1): med |
| *EGFR* (2) | EGFR inhibitor (2) | EGFR inhibitor (2): high |
| *ERBB2* (1) | ERBB2 inhibitor (1) | ERBB2 inhibitor (1): med |
| *FBXW7* (5) |  | MTORC1 inhibitor (5): med |
| *GNAS* (1) | Hedgehog inhibitor (1) |  |
| *HRAS* (2) | MEK inhibitor (1) | MAP2K2/MAP2K1 inhibitor (2): med  MAPK1/MAPK3 inhibitor (1): low |
| *IDH1* (1) |  | IDH1 inhibitor (1): med |
| *KRAS* (15) | CDK4/6 inhibitors (2)  ERK inhibitor (1)  MEK inhibitors (3)  RAF inhibitor (1) | MAP2K2/MAP2K1 inhibitor (15): med  MAPK1/MAPK3 inhibitor (2): low |
| *MAP2K1* (1) | MEK inhibitor (1) | MAP2K2/MAP2K1 inhibitor (1): low |
| *NF1* (5) | MEK inhibitor (5)  mTOR inhibitor (2) | MAP2K2/MAP2K1 inhibitor (5): med  MAPK1/MAPK3 inhibitor (3): low |
| *NRAS* (1) |  | MAP2K2/MAP2K1 inhibitor (1): med |
| *PDGFRA* (1) | PDGFR inhibitor (1) | PDGFR inhibitor (1): high |
| *PIK3CA* (19) | PIK3CA inhibitors (19)  AKT inhibitors (19)  mTOR inhibitor (2) | PIK3CA inhibitor (19): med  MTORC1 inhibitor (19): med-high |
| *PTCH1* (2) | Hedgehog inhibitor (1) | SMO inhibitor (2): med |
| *PTEN* (2) | AKT inhibitor (1)  mTOR inhibitor (1) | PIK3CB inhibitor (2): med  MTORC1 inhibitor (2): med  PARP inhibitor (2): med |
| *STK11* (1) |  | MTORC1 inhibitor (1): med |
| *TP 53* (80) |  | WEE1 inhibitor (80): med  TP 53 gene therapy (80): med |
| *TSC1* (1) | mTOR inhibitor (1) | MTORC1 inhibitor (1): high |
| Amplification |  |  |
| *MYC* (10) | PIM inhibitor (6) | CDK1/2 inhibitor (10):low  AURKA inhibitor (10): low  AURKB inhibitor (2): low |
| *ERBB2* (8) | ERBB2 inhibitor (8) | ERBB2 inhibitor (8): high |
| *CCND1* (6) | CDK4/6 inhibitor (6) | CDK4/6 inhibitor (6): high |
| *MDM2* (4) | MDM2 inhibitor (4) | TP53 gene therapy (3): low |
| *EGFR* (4) | EGFR inhibitor (4) | EGFR inhibitor (4) : high |
| *FGFR1* (2) | FGFR inhibitor (2) | FGFR 1 inhibitor (2): med |
| *CDK4* (1) | CDK4/6 inhibitor (1) | CDK4/6 inhibitor (1): high |
| *IGF1R* (1) | IGF1R inhibitor (1) | IGF1R inhibitor (1): low |
| *CD274* (1) | PD-1 inhibitor (1) | PD-1 inhibitor (1) : med |
| Fusions |  |  |
| *CLTC-ALK* | ALK inhibitor (1) | ALK inhibitor (1): high |
| *GBA3-ALK* | ALK inhibitor (1) | ALK inhibitor (1): high |
| *KIF5B-RET* | RET inhibitor (1) | RET inhibitor (1): high |
| *CD74-ROS1* | ROS1 inhibitor (1) | ROS1 inhibitor (1): high |
| “high”, “med”, and “low” mean the potential degree of each targeted therapy. | | |
