# Supplementary material for: Evaluating Clinical Genome Sequence Analysis by Watson for Genomics
Source: Front Med (Lausanne). 2018 Nov 9;5:305. doi: 10.3389/fmed.2018.00305 (PMC6237914; doi:10.3389/fmed.2018.00305)
Supplement: Supplementary file 10 [file Data_Sheet_1.PDF]

IBM **Watson** for **Genomics**

Version 27.87

REPORT FOR: **National Cancer Center**

[redacted] [redacted]

CASE #: [redacted] SAMPLE #: [redacted]

TERMS OF USE

IBM Watson for Genomics searches electronic medical databases to find information that may be relevant to a particular genomic sequence. IBM Watson for Genomics is a research tool. You remain responsible for the conduct of your patient care and for evaluating the clinical relevance of the information provided by the tool. You agree that any use of the tool by you does not diminish your responsibility for patient care. In providing you with access to the tool, IBM is acting solely as a services provider and does not purport to be engaged in the practice of medicine or any other professional clinical or licensed activity, and the tool consists solely of support to facilitate providing information to you. You must evaluate the safety and security of the tool and the reports it generates, and exercise of your independent professional judgment regarding any care decisions resulting from your use. IBM assumes no responsibility or liability for the accuracy, completeness, propriety, necessity, security or advisability of the tool or the reports and any medical information which is accessed through the use of the tool or the reports. You understand that the tool contains information based on and generated for use in the United States; there is no information specific to any other country in the tool.

(c) Copyright IBM Corp. 2014, 2016 All Rights Reserved. US Government Users Restricted Rights - Use, duplication or disclosure restricted by GSA ADP Schedule Contract with IBM Corp. Licensed Materials - Property of IBM. IBM, the IBM logo, and IBM Watson are trademarks of IBM Corporation, registered in many jurisdictions worldwide.

IBM **Watson** for **Genomics** Version 27.87

National Cancer Center
CASE #: [redacted]
SAMPLE #: [redacted]

SUMMARY

DIAGNOSIS:
Invasive Ductal Carcinoma

3	Actionable Alterations	4	FDA Approved for Invasive Ductal Carcinoma	7	Therapies with Clinical Trial(s)	0	FDA Approved for Other Cancer(s)
**ERBB2**	*Amplification*	Lapatinib<br>Pertuzumab<br>Trastuzumab<br>Trastuzum...	Level 1<br>Level 1<br>Level 1<br>Level 1	**Phase 2**<br>1 Level 3A<br>**Phase 1**<br>2 Level 4		-	
**MYC**	*Amplification*	-		**Phase 1**<br>2 Level 4		-	
**TP53**	*R342fs*	-		**Phase 2**<br>1 Level 3B<br>**Phase 1**<br>1 Level 3B		-	

For the complete molecular diagram see last page.

Copyright IBM Corp. 2014, 2016

ACTIONABLE ALTERATIONS WITH THERAPIES

ERBB2	*Amplification*	4	FDA Approved for Invasive Ductal Carcinoma	3	Therapies with Clinical Trial(s)	0	FDA Approved for Other Cancer(s)

Gene Summary: ERBB2 is a member of the EGF receptor family of receptor tyrosine kinases. Unlike other members, it does not have a ligand binding domain and cannot bind growth factors. However, it has the ability to undergo ligand-independent dimerization and binds to other ligand-bound EGF receptor family members to form a heterodimer. Dimerization results in the autophosphorylation of tyrosine residues within the cytoplasmic domain of the receptors and initiates signaling pathways that link to cell proliferation. ERBB2 overexpression induces constitutive activity, and the gene is amplified or overexpressed in up to 20% of breast cancers, correlating with poor survival. Overexpression of ERBB2 is also seen other malignancies, most commonly in gastric adenocarcinoma, adenocarcinoma of the esophagus and gastro-esophageal junction, CRC, and endometrial carcinoma. Activation of ERBB2 signaling, either through ERBB2 amplification or through heregulin up-regulation, leads to persistent extracellular signal-regulated kinase 1/2 signaling and consequently to cetuximab resistance and amplification has been identified as cause of resistance to therapy with the anti-EGFR antibody cetuximab in colorectal cancer (PubMed: 26030179).

CNV Amplification: ERBB2 amplification refers to when multiple copies of the segment of the genome harboring the ERBB2 gene are aberrantly generated in the cell, underlying pathogenic overexpression of the encoded ERBB2 protein (reviewed in (PubMed: 24656976)). ERBB2 amplification and associated protein overexpression increases signaling through the oncogenic and catabolic RAS/MAPK, PI3K/AKT/MTOR, SRC and STAT pathways (PubMed: 23204226, 12124352), and is a transforming event (PubMed: 2885917, 10716706, 11571643).

Lapatinib - *LEVEL 1*

TARGET: **ERBB2**

FDA Approved for Invasive Ductal Carcinoma

FDA Label: In combination with capecitabine for the treatment of patients with advanced or metastatic breast cancer whose tumors overexpress HER2 and who have received prior therapy including an anthracycline, a taxane, and trastuzumab. Limitation of Use: Patients should have disease progression on trastuzumab prior to initiation of treatment with TYKERB in combination with capecitabine. In combination with letrozole for the treatment of postmenopausal women with hormone receptor-positive metastatic breast cancer that overexpresses the HER2 receptor for whom hormonal therapy is indicated.

Pertuzumab - *LEVEL 1*

TARGET: **ERBB2**

FDA Approved for Invasive Ductal Carcinoma

FDA Label: In combination with trastuzumab and docetaxel for the treatment of patients with HER2-positive metastatic breast cancer who have not received prior anti-HER2 therapy or chemotherapy for metastatic disease and in combination with trastuzumab and docetaxel for the neoadjuvant treatment of patients with HER2-positive, locally advanced, inflammatory or early-stage breast cancer (either greater than 2 cm in diameter or node positive) as part of a complete treatment regimen for early breast cancer.

Trastuzumab - *LEVEL 1*

TARGET: **ERBB2**

FDA Approved for Invasive Ductal Carcinoma

Copyright IBM Corp. 2014, 2016

FDA Label: As part of a treatment regimen containing doxorubicin, cyclophosphamide, and paclitaxel for the adjuvant treatment of women with node-positive, HER2-overexpressing breast cancer. In combination with cisplatin and capecitabine or 5-fluorouracil, for the treatment of patients with HER2 overexpressing metastatic gastric or gastroesophageal junction adenocarcinoma, who have not received prior treatment for metastatic disease.

Trastuzumab Emtansine - *LEVEL 1*

TARGET: **ERBB2**

FDA Approved for Invasive Ductal Carcinoma

FDA Label: For the treatment of patients with HER2-positive, metastatic breast cancer who previously received trastuzumab and a taxane, separately or in combination.

Neratinib (PB272 HKI-272) - *LEVEL 3A*

TARGET: **ERBB2**

Therapy with Clinical Trial(s)

Mechanism of Action: An orally available, 6,7-disubstituted-4-anilinoquinoline-3-carbonitrile irreversible inhibitor of the HER-2 receptor tyrosine kinase with potential antineoplastic activity. Neratinib binds to the HER-2 receptor irreversibly, thereby reducing autophosphorylation in cells, apparently by targeting a cysteine residue in the ATP-binding pocket of the receptor. Treatment of cells with this agent results in inhibition of downstream signal transduction events and cell cycle regulatory pathways; arrest at the G1-S (Gap 1/DNA synthesis)-phase transition of the cell division cycle; and ultimately decreased cellular proliferation. Neratinib also inhibits the epidermal growth factor receptor (EGFR) kinase and the proliferation of EGFR-dependent cells.

NCT #	Clinical Trials	Phase
NCT02673398	Phase II Study of Neratinib in Patients 60 and Older With HER2 Positive Locally Advanced or Metastatic Breast Cancer	2<br>Not yet recruiting
NCT01494662	A Phase II Trial of HKI-272 (Neratinib) and Capecitabine for Patients With Human Epidermal Growth Factor Receptor 2 (HER2)-Positive Breast Cancer and Brain Metastases	2
NCT02236000	A Phase Ib/II Dose-Escalation Study Evaluating the Combination of Trastuzumab Emtansine (T-DM1) With Neratinib in Women With Metastatic HER2-Positive Breast Cancer	1 or 2

Afatinib - *LEVEL 4*

TARGET: **ERBB2**

Therapy with Clinical Trial(s)

Copyright IBM Corp. 2014, 2016

Mechanism of Action: An orally bioavailable anilino-quinazoline derivative and inhibitor of the receptor tyrosine kinase (RTK) epidermal growth factor receptor (ErbB; EGFR) family, with antineoplastic activity. Upon administration, afatinib selectively and irreversibly binds to and inhibits the epidermal growth factor receptors 1 (ErbB1; EGFR), 2 (ErbB2; HER2), and 4 (ErbB4; HER4), and certain EGFR mutants, including those caused by EGFR exon 19 deletion mutations or exon 21 (L858R) mutations. This may result in the inhibition of tumor growth and angiogenesis in tumor cells overexpressing these RTKs. Additionally, afatinib inhibits the EGFR T790M gatekeeper mutation which is resistant to treatment with first-generation EGFR inhibitors. EGFR, HER2 and HER4 are RTKs that belong to the EGFR superfamily; they play major roles in both tumor cell proliferation and tumor vascularization and are overexpressed in many cancer cell types.

NCT #	Clinical Trials	Phase
NCT02451553	Phase I/IB Multi-center Study of Irreversible EGFR/HER2 Tyrosine Kinase Inhibitor Afatinib (BIBW 2992) in Combination With Capecitabine for Advanced Solid Tumors and Pancretico-Biliary Cancers	1

Dacomitinib (PF-00299804) - *LEVEL 4*

TARGET: **ERBB2**

Therapy with Clinical Trial(s)

Mechanism of Action: A highly selective, orally bioavailable small-molecule inhibitor of the HER family of tyrosine kinases with potential antineoplastic activity. Dacomitinib specifically and irreversibly binds to and inhibits human Her-1, Her-2, and Her-4, resulting in the proliferation inhibition and apoptosis of tumor cells that overexpress these receptors.

NCT #	Clinical Trials	Phase
NCT01920061	A Phase 1b Open-Label Three-Arm Multi-Center Study To Assess The Safety And Tolerability Of PF-05212384 ((PI3K/mTOR Inhibitor) In Combination With Other Anti-Tumor Agents	1

MYC	*Amplification*	0	FDA Approved for Invasive Ductal Carcinoma	2	Therapies with Clinical Trial(s)	0	FDA Approved for Other Cancer(s)

Gene Summary: MYC is a multifunctional nuclear phosphoprotein and transcriptional regulator with roles in cell cycle progression, proliferation, differentiation and apoptosis. The protein functions as a transcription factor that regulates transcription of specific target genes. It carries a common basic-helix-loop-helix leucine zipper (bHLH-ZIP) motif required for dimerization and DNA-binding. MYC associates with MAX, another member of the basic helix-loop-helix leucine zipper (bHLHZ) family of transcription factors resulting in DNA binding and activation of transcription. The association between MAX and either MYC can have opposing effects on transcriptional regulation and cell behavior. MYC has been shown to promote tumorigenesis by inducing proliferation, inhibiting exit from the cell cycle, stimulation of vascularization and by enhancing genomic instability (PubMed: 10378696, 16934487, 19029958, 22464321). Activation in cancer can occur through a variety of mechanisms, including chromosomal translocations in hematological malignancies. The most common alteration associated with solid tumors is amplification, while missense mutations are less frequent.

 Copyright IBM Corp. 2014, 2016

CNV Amplification: Amplification of MYC is correlated with an increase in the expression of the MYC gene (PubMed: 15083194, 22464321), which leads to the transcriptional activation of MYC target genes. MYC target genes regulate various cellular processes involved in oncogenesis including proliferation, inhibiting exit from the cell cycle, stimulating vascularization and enhancing genomic instability (PubMed: 10378696, 16934487, 19029958, 22464321). Genomic amplification of MYC is observed in a large number of tumor types. A study of genomic amplification across many cancer types identified focal amplifications of MYC in 14% of all cancers, with additional enrichment for large scale arm-level copy number alterations encompassing the MYC gene locus (PubMed: 20164920).

Alisertib (MLN8237) - *LEVEL 4*

TARGET: **AURKA**

Therapy with Clinical Trial(s)
**Mechanism of Action:** A second-generation, orally bioavailable, highly selective small molecule inhibitor of the serine/threonine protein kinase Aurora A kinase with potential antineoplastic activity. Alisertib binds to and inhibits Aurora A kinase, which may result in disruption of the assembly of the mitotic spindle apparatus, disruption of chromosome segregation, and inhibition of cell proliferation. Aurora A kinase localizes to the spindle poles and to spindle microtubules during mitosis, and is thought to regulate spindle assembly. Aberrant expression of Aurora kinases occurs in a wide variety of cancers, including colon and breast cancers.

NCT #	Clinical Trials	Phase
NCT02327169	A Multiarm, Open-label, Phase 1b Study of MLN2480 (an Oral A-, B-, and CRAF Inhibitor) in Combination With MLN0128 (an Oral mTORC 1/2 Inhibitor), or Alisertib (an Oral Aurora A Kinase Inhibitor), or Paclitaxel, or Cetuximab, or Irinotecan, in Adult Patients With Advanced Nonhematologic Malignancies	1

Dinaciclib (SCH-727965) - *LEVEL 4*

TARGET: **CDK1**

Therapy with Clinical Trial(s)
**Mechanism of Action:** A pyrazolo[1,5-a]pyrimidine with potential antineoplastic activity. Dinaciclib selectively inhibits cyclin dependent kinases CDK1, CDK2, CDK5, and CDK9; inhibition of CDK1 and CDK2 may result in cell cycle repression and tumor cell apoptosis.

NCT #	Clinical Trials	Phase
NCT01434316	Phase 1 Trial of ABT-888 and SCH727965 in Patients With Advanced Solid Tumors	1

**TP53**	*R342fs*	**0**	FDA Approved for Invasive Ductal Carcinoma	**2**	Therapies with Clinical Trial(s)	**0**	FDA Approved for Other Cancer(s)

Copyright IBM Corp. 2014, 2016

Gene Summary: The transcription factor TP53 regulates a large number of genes that control tumor suppressing functions such as cell cycle arrest, DNA repair, senescence and apoptosis, whilst the activation of TP53 often leads to apoptosis. Protein levels and post-translational modification state alter in response to cellular stress (such as DNA damage, hypoxia, spindle damage). Activation of TP53 begins through a number of mechanisms including phosphorylation by ATM, ATR, CHK1 and MAPKs. The ubiquitin ligase MDM2 binds to and targets TP53 for proteasomal degradation and preventing this interaction results in an increase of stable TP53 tetramers in the cytoplasm. Modifications such as methylation and acetylation lead to an increase in TP53 binding to gene specific response elements. The TP53 tumor suppressor gene regulates more than 100 genes that control critical tumor suppressing functions such as cell cycle arrest, DNA repair, senescence and apoptosis. It is the most frequently altered gene in human cancers and missense mutations that are associated with an aggressive phenotype occur in more than 50% of cancers.

Inactivating Mutation: Truncating mutations, which disrupt the DNA-binding domain or oligodimerization domain of the protein are predicted to be inactivating (PubMed: 16007150, 11900253, 11753428). Inactivation of TP53 can confer cells additive growth and survival advantages, such as increased proliferation, evasion of apoptosis, and chemoresistance (PubMed: 19691397, 11156366).

R342fs: This mutation is an inactivating mutation, thus it is classified as pathogenic.

AZD1775 - *LEVEL 3B*

TARGET: **WEE1**

Therapy with Clinical Trial(s)
**Mechanism of Action:** A small molecule inhibitor of the tyrosine kinase WEE1 with potential antineoplastic sensitizing activity. AZD1775 selectively targets and inhibits WEE1, a tyrosine kinase that phosphorylates cyclin-dependent kinase 1 (CDK1, CDC2) to inactivate the CDC2/cyclin B complex. Inhibition of WEE1 activity prevents the phosphorylation of CDC2 and impairs the G2 DNA damage checkpoint. This may lead to apoptosis upon treatment with DNA damaging chemotherapeutic agents. Unlike normal cells, most p53 deficient or mutated human cancers lack the G1 checkpoint as p53 is the key regulator of the G1 checkpoint and these cells rely on the G2 checkpoint for DNA repair to damaged cells. Annulment of the G2 checkpoint may therefore make p53 deficient tumor cells more vulnerable to antineoplastic agents and enhance their cytotoxic effect.

NCT #	Clinical Trials	Phase
NCT02576444	A Phase II Study of the PARP Inhibitor Olaparib (AZD2281) Alone and in Combination With AZD1775, AZD5363, or AZD2014 in Advanced Solid Tumors	2
NCT02095132	A Phase 1/2 Study of AZD1775 (MK-1775) in Combination With Oral Irinotecan in Children, Adolescents, and Young Adults With Relapsed or Refractory Solid Tumors	1 or 2
NCT02610075	A Phase Ib Study to Determine the Maximum Tolerated Dose (MTD) of AZD1775 Monotherapy in Patients With Locally Advanced or Metastatic Solid Tumours.	1
NCT01748825	A Phase I Study of Single-agent AZD1775 (MK-1775), a Wee1 Inhibitor, in Patients With Advanced Refractory Solid Tumors	1

Copyright IBM Corp. 2014, 2016

NCT02511795	A Phase Ib Study of AZD1775 and Olaparib in Patients With Refractory Solid Tumours	1
NCT02617277	A Phase I Study Assessing the Safety, Tolerability and Pharmacokinetics of AZD1775 in Combination With MEDI4736 in Patients With Advanced Solid Tumours	1

Transferrin Receptor-Targeted Liposomal p53 cDNA - *LEVEL 3B*

TARGET: **TP53**

Therapy with Clinical Trial(s)
**Mechanism of Action:** A cationic liposomal, tumor-targeting p53 (TP53) gene delivery system with potential anti-tumor activity. Transferrin receptor-targeted liposomal p53 cDNA contains plasmid DNA encoding the tumor suppressor protein p53 packaged in membrane-like liposome capsules that are complexed with anti-transferrin receptor single-chain antibody (TfRscFv). Upon systemic administration, the anti-TfRscFv selectively binds to tumor cells expressing transferrin receptors. The p53 plasmid is delivered into the nucleus and as a result, p53 protein is produced in tumor cells that have altered p53 function. This results in the restoration of normal cell growth control mechanisms as well as normal response mechanisms to DNA damage.

NCT #	Clinical Trials	Phase
NCT02354547	A Phase I Study of SGT-53, a TfRscFv-Liposome-p53 Complex, in Children With Refractory or Recurrent Solid Tumors	1

Copyright IBM Corp. 2014, 2016

ALTERATIONS WITHOUT THERAPIES

NOTCH3 *Amplification*

Gene Summary: NOTCH3 is a member of the NOTCH family of transmembrane proteins and transcription factors. It is the receptor for membrane-bound ligands Jagged1, Jagged2 and Delta1 and regulates cell-fate determination and maturation. Unlike the wildly expressed NOTCH1 and -2 receptors, NOTCH3 expression is largely restricted to vascular smooth muscle cells (PubMed: 10712431, 11578869), to the central nervous system (PubMed: 7918097), a subset of thymocytes (PubMed: 10383933), and regulatory T cells (PubMed: 14568923). Constitutive activation of NOTCH3 as a consequence of mutations has been shown to result in tumorigenesis (PubMed: 10880446). Several independent lines of study have linked NOTCH3 to lung cancer by overexpression (PubMed: 10944559, 17804716), through a critical, non-redundant role in the self-renewal of tumor-propagating cells (PubMed: 23845442), and to poor overall patient survival through activation in the ALDH (aldehyde dehydrogenase) positive cell population, which has been associated with stem-like cell characteristics conferring the ability of colony and tumor formation (PubMed: 23444212). Mutations in the PEST domain, resulting in compromised protein degradation, have been identified in triple negative breast cancer(PubMed: 25564152), while amplification of the gene has been described in 12% of serous ovarian carcinoma (PubMed: 21720365).

 Copyright IBM Corp. 2014, 2016

APPENDIX A

VARIANTS OF UNKNOWN SIGNIFICANCE

Note: One or more variants of unknown significance (VUS) were detected in this patient’s tumor. These variants have not been adequately characterized in the scientific literature and possible effects on protein function and pathological significance are unknown. We choose to include them in this report in the event that their clinical relevance is determined in the future.

ERBB4	*T422I*	KEAP1	*G571D*

Copyright IBM Corp. 2014, 2016

APPENDIX B

POTENTIAL RESISTANCE IN THESE THERAPIES
**<None>**

 Copyright IBM Corp. 2014, 2016

APPENDIX C

LEVELS OF EVIDENCE

Level	Description
1	**1 -** FDA approved drug in this cancer type and biomarker
2A	**2A -** Standard of care biomarker predictive of response to an FDA approved drug in this indication
2B	**2B -** Standard of care biomarker predictive of response to an FDA approved drug in a different indication
3A	**3A -** Compelling clinical evidence supports the biomarker as being predictive of response to a drug in this indication
3B	**3B -** Compelling clinical evidence supports the biomarker as being predictive of response to a drug in a different indication
4	**4 -** Compelling biological evidence supports the biomarker as being predictive of response to a drug
R1	**R1 -** Standard of care biomarker predictive of resistance to an FDA approved drug in this indication
R2	**R2 -** Includes drugs with compelling clinical evidence that support the biomarker as being predictive of resistance to a drug in this indication.

Copyright IBM Corp. 2014, 2016

APPENDIX D

MOLECULAR PROFILE DIAGRAM

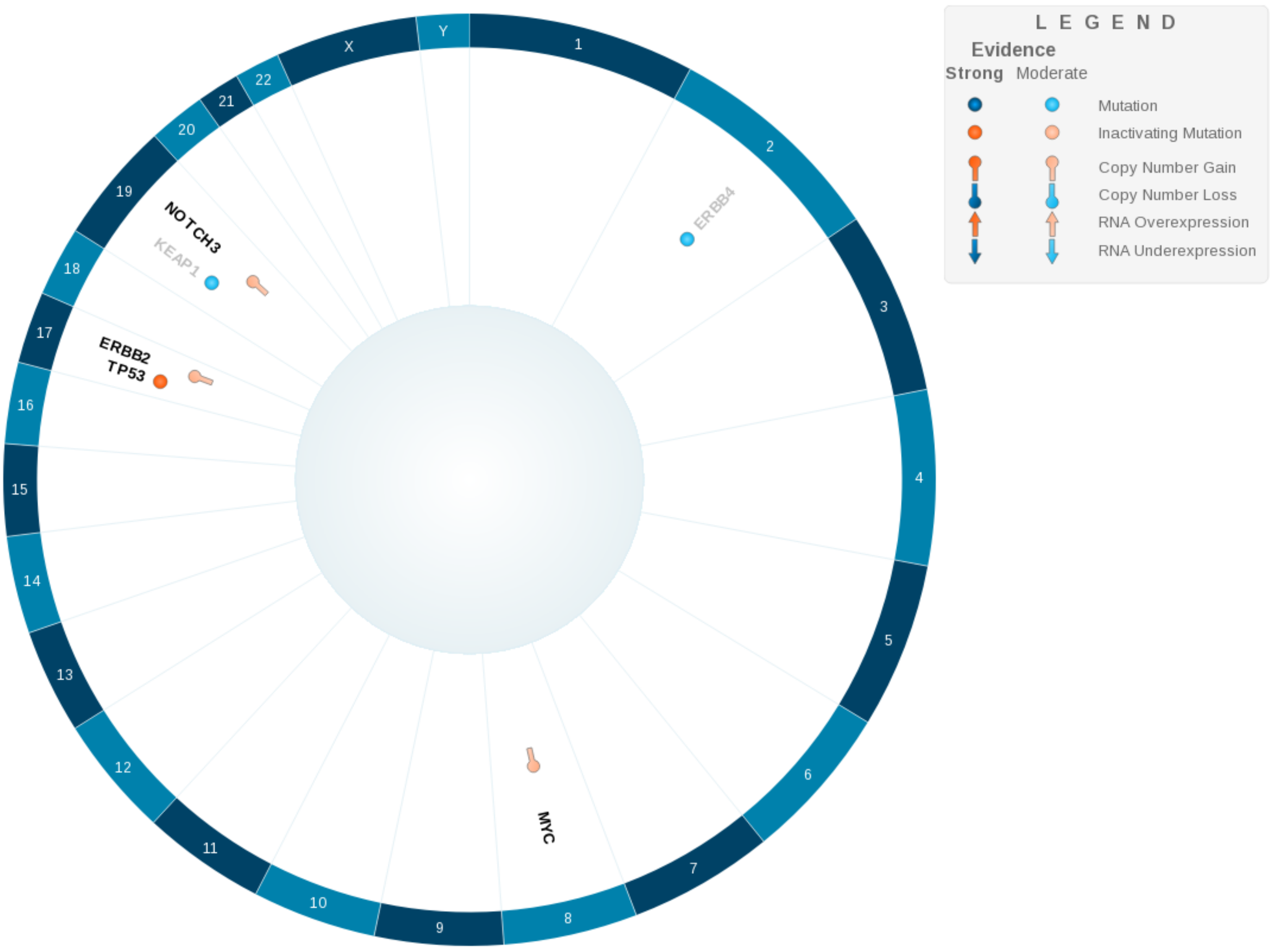

END OF REPORT

Copyright IBM Corp. 2014, 2016
